# Strong interface scattering induced low thermal conductivity in Bi-based GeTe/Bi_2_Te_3_ superlattice-like materials

**DOI:** 10.1039/c9ra01485c

**Published:** 2019-03-25

**Authors:** Yang Zhou, Kaijin Huang, Lingjun Zhou, Xiaomin Cheng, Ming Xu, Hao Tong, Xiangshui Miao

**Affiliations:** Wuhan National Laboratory for Optoelectronics, Huazhong University of Science and Technology Wuhan 430074 China tonghao@hust.edu.cn

## Abstract

The thermal conductivities of GeTe/Bi_2_Te_3_ superlattice-like materials are calculated based on density functional perturbation theory (DFPT) and measured using a 3ω method. The calculated results show that the lattice thermal conductivity or thermal diffusivity of GeTe/Bi_2_Te_3_ superlattice-like materials significantly decrease due to the effects of interfaces and Bi atoms in Bi_2_Te_3_. Our measured results are in line with the theoretical calculations, and reach an extremely low thermal conductivity at 0.162 W mK^−1^ compared with published work on Ge–Sb(Bi)–Te, indicating the effectiveness of modulating the thermal properties of phase change materials by using Bi-based GeTe/Bi_2_Te_3_ superlattice-like materials. Our findings give a calculation method to modify the thermal characteristics of superlattice-like materials and confirm Bi-based GeTe/Bi_2_Te_3_ superlattice-like materials as promising candidates for phase change materials with lower thermal conductivity.

## Abstract 2

Strong interface scattering caused by Bi atoms in Bi-based GeTe/Bi_2_Te_3_ superlattice-like materials leads to the decrease of thermal conductivity.

## Graphical abstract

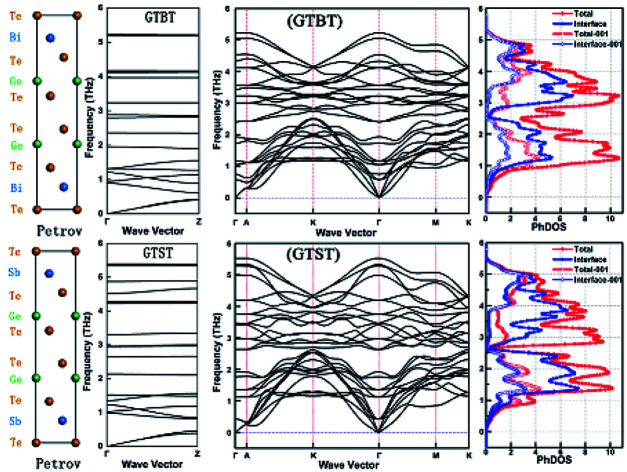

## Introduction

Phase change memory^1^ (PCM) is a promising candidate for next-generation nonvolatile memory due to its low power consumption, high endurance, and high speed. Apart from accelerating the phase transition speed and lowering the power consumption,^2^ phase-change materials with a low thermal conductivity are of crucial importance to reduce programming current^3^ and suppress the thermal crosstalk between adjacent storage units,^4^ which are key issues for PCM in high-density integration and scaling technology.^5,6^ Considering the important influence of thermal conductivity, we investigate the possibility of whether there is any efficient way of decreasing in thermal conductivity of phase-change materials to improve PCM technology. Ge_2_Sb_2_Te_5_ has been extensively studied and widely used in phase change memories for its excellent performance,^7^ and it can be considered as the combination of GeTe and Sb_2_Te_3_. The property enhancement has been achieved in Ge_2_Sb_2_Te_5_ two-component phase-change superlattice structures,^3^ and the thermal conductivity of superlattice-like materials is considerably lower than that of the constituent bulk materials.^8^ Since the component materials and the interfaces between two components being main factors of the thermal conductivity in superlattice materials, one of the ways to control two-component phase-change superlattice materials properties is to choose the appropriate chalcogenide materials like Bi_2_Te_3_ for weakening the cohesion and enhancing the crystallization by bismuth.^9^ However, the accurate thermal conductivity of GeTe/Bi_2_Te_3_ superlattice materials has not been reported anywhere and how the Bi atoms of GeTe/Bi_2_Te_3_ superlattice structures influence on interfaces and modulate the thermal conductivity are totally unknown. In this letter, we implement the theoretical calculations to investigate the lattice thermal conductivity of GeTe/Bi_2_Te_3_ superlattice-like materials, and show a calculation method to modify thermal characteristics of superlattice-like materials for better performance.

### Model structures and simulation methodology

The molecular dynamics (MD) simulations are performed using the Vienna ab initio simulation package^10^ (VASP) in this letter. Superlattice structure models were first relaxed using density functional theory (DFT) within generalized gradient approximations of Perdew–Burke–Ernzerhof (PBE) in conjunction with projector augmented wave (PAW) potentials. The relaxation convergences for ions and electrons were 1 × 10^−7^ and 1 × 10^−8^ eV, respectively. The automatically generated *k*-points mesh of 7 × 7 × 2 with Gamma symmetry was used. The energy cut-off is 400 eV.

Bi_2_Te_3_ has rhombohedral crystal structure belonging to the space group *R*3*m* with five atoms in one unit cell, the lattice parameters of the five-layer hexagonal unit cell built up by three formula units are *a* = *b* = 4.384 Å, *c* = 30.497 Å, and *α* = *β* = 90°, *γ* = 120°.^11^ For GeTe, a distorted rocksalt structure with *a* = *b* = *c* = 5.996 Å and *α* = *β* = *γ* = 90°, belonging to space group *R*3*m*.^12^ The crystal structures of the GTBT (GeTe/Bi_2_Te_3_) superlattices were constructed from the <001> direction of trigonal Bi_2_Te_3_ and the <111> direction of cubic GeTe, thus superlattices exhibit high quality interfaces due to the good lattice parameter match. We consider that the interfacial state and the strength of the Bi atoms' influence on interfaces may have effect on the thermal conductivity. In order to support the conjecture, we choose four common superlattice structures with different number of interfaces or different distance between Bi atoms and interfaces. Table 1 lists the lattice constants of the four GTBT superlattice structures with the Petrov,^13^ inverted Petrov,^14^ Kooi, and Ferro-GeTe^15^ phase using PBE. Additionally, the features of superlattice structures, including the number of the long distance Te–Te interfaces (*N*_i_) and the number of Bi atoms near to the interfaces (*N*_n_), are also shown. Meanwhile, GTST (GeTe/Sb_2_Te_3_) as common superlattice materials are also listed to providing comprehensive analysis of the thermal property change trend with GTBT superlattices in same structures, and the lattice parameters of the five-layer hexagonal unit cell in Sb_2_Te_3_ are *a* = *b* = 4.264 Å, *c* = 30.458 Å, and *α* = *β* = 90°, *γ* = 120°. For both GTBT and GTST superlattice structures, the lattice parameter *a* = *b*, and *α* = 90°, *β* = 90°, *γ* = 120°.

Lattice constants for GTBT and GTST superlattice structures with the Petrov, inverted Petrov, Kooi, and Ferro-GeTe phase by PAW-PBE calculations

**Table:** 

	Inverted Petrov				Petrov			
	*a* (*b*) (Å)	*c* (Å)	*N*_i_	*N*_n_	*a* (*b*) (Å)	*c* (Å)	*N*_i_	*N*_n_
GTST	4.205	19.405	2	2	4.266	17.982	1	0
GTBT	4.269	19.445			4.327	18.143

**Table:** 

	Ferro-GeTe				Kooi			
	*a* (*b*) (Å)	*c* (Å)	*N*_i_	*N*_n_	*a* (*b*) (Å)	*c* (Å)	*N*_i_	*N*_n_
GTST	4.283	17.677	1	1	4.293	17.632	—	—
GTBT	4.341	17.893			4.353	17.934		

**Fig. 1:**
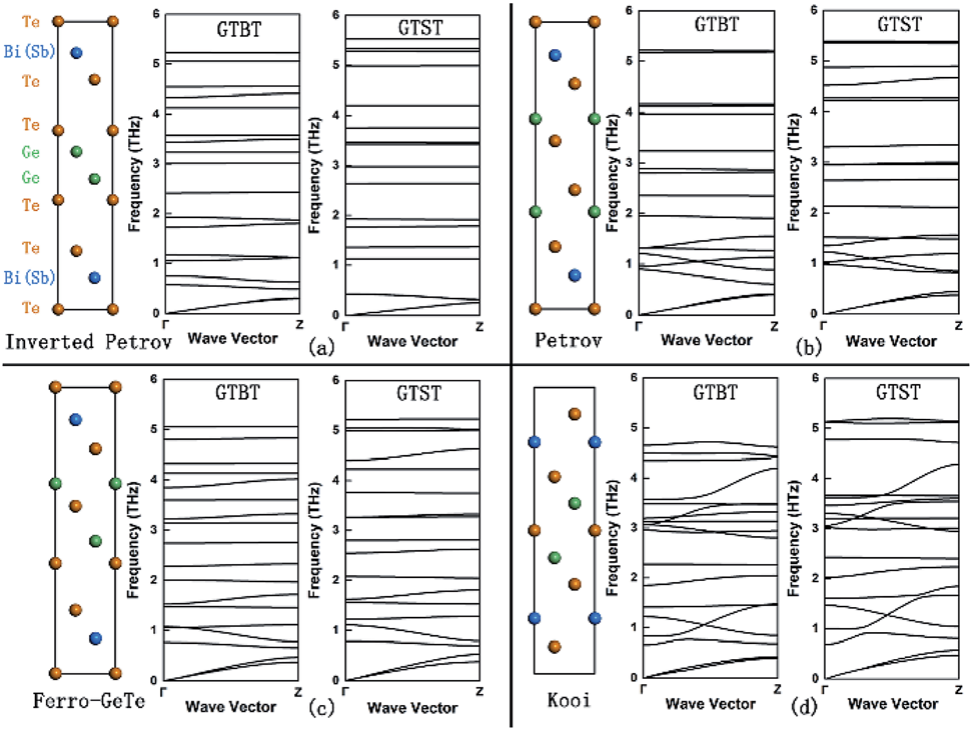
The structures of phase-change superlattice with the inverted Petrov (a), Petrov (b), Ferro-GeTe (c) and Kooi (d) phase. Next to each structure, the corresponding phonon dispersion curves along the out-of-plane direction (Γ- to Z-direction) are shown, for GTST and GTBT, respectively.

The crystal structures of GTBT superlattice are illustrated in Fig. 1, and the corresponding phonon dispersion curves along the out-of-plane direction (Γ–Z direction) are shown in the right column. The PHONOPY code^16^ was performed to calculate the phonon frequencies through a supercell method. In calculating the force constants in real space we use density functional perturbation theory (DFPT) together with 72 atoms 2 × 2 × 2 supercells, the phonon band structure and density of states are generated from the force constants.

### Role of interfaces and Bi atoms in thermal conductivity

The superlattice materials in Fig. 1 show very flat phonon dispersion curves, whose flatness are obviously higher than the mono-composition materials GeTe,^17^ Bi_2_Te_3_.^18^ Since the group velocity *V*_g_ = d*ω*/d*q*, GTBT superlattices have a sharply decrease in phonon group velocity compared to single layer material. This significantly low group velocity arises from the interface phonon scattering and phonon confinement effects.^19,20^ Therefore, the impact of interfaces impact could explain the flat phonon curves and the low thermal conductivity of the superlattice materials across the interface. We worked out that the order of group velocity of GTBT is as follows: inverted Petrov < Ferro-GeTe < Petrov < Kooi phase. What is more, the structure features of the superlattices, *N*_i_ and *N*_n_, are also decreasing in the same order, which have been listed in Table 1. This result indicates that: as the number the long distance Te–Te interfaces or the number of Bi atoms near to the interfaces decreases, the strength of interfaces' effect becomes weaker and leads to decreased flatness of phonon dispersion curves in sequence, which results in decreasing trend of group velocity. Meanwhile, we noticed that GTBT superlattices have more flat phonon dispersion curves along the out-of-plane direction (Γ–Z direction) comparing to GTST superlattices, resulting in a smaller phonon group velocity. This phenomenon verifies strong interface scattering in GTBT superlattices because of the Bi atoms' impact on interface.

**Fig. 2:**
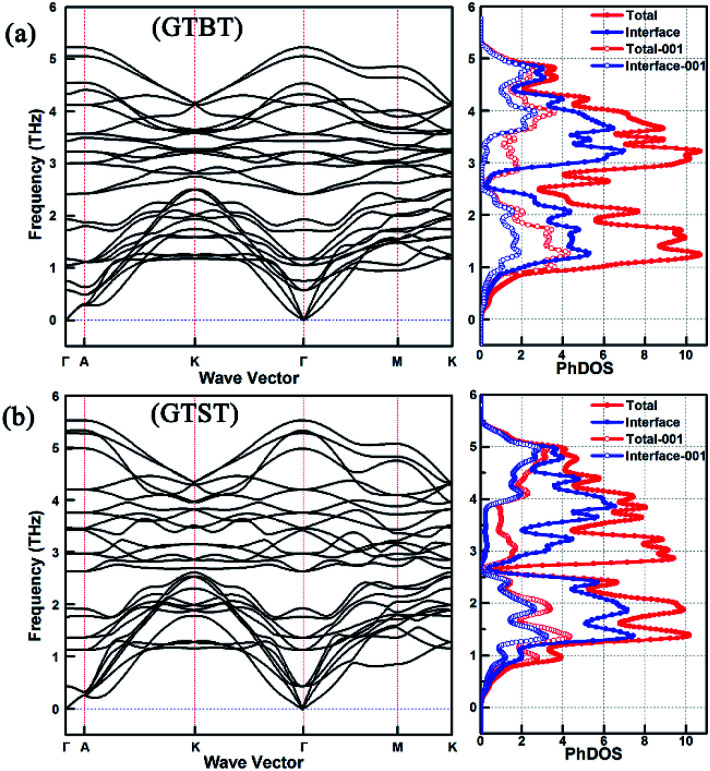
Phonon dispersion curves for (a) GTBT, (b) GTST with inverted Petrov phase along the Γ–A–K–Γ–M–K direction, next to each phonon dispersion curve, the corresponding total PhDOS, partial PhDOS due to interface (containing two Ge atoms, four Te atoms), and their partial PhDOS along the <001 > direction are shown.

For further studying the source of the significant small group velocity in GTBT superlattice structures, we calculated the phonon dispersion curves and the PhDOS of superlattice structures. The result of superlattice structures with inverted Petrov phase were chosen as an example to discussed. As shown in Fig. 2, we plot the calculated phonon dispersion curves along the Γ–A–K–Γ–M–K direction for GTBT superlattices with inverted Petrov phase. Next to each phonon dispersion curves, the corresponding total PhDOS, partial PhDOS due to interface (containing two Ge atoms, four Te atoms), and their partial PhDOS along the <001 > direction are shown. Fig. 2 shows that compared with the total PhDOS, the contribution rate of GTBT superlattice structure's partial PhDOS due to interface is significantly small in lower frequencies range, the same phenomenon can be found along the <001 > direction, these obviously indicate Bi atoms' impact on interface and the strong interface scattering of GTBT, which would result in small phonon group velocity and finally bring decrease in lattice thermal conductivity. Meanwhile, the result shows the Γ–A direction, owing ultra-flattened phonon dispersion curves, differs significantly from the other directions, this indicates the anisotropic phonon group velocity of superlattice materials, and ultimately results in anisotropic lattice thermal conductivity. Additionally, we can find the PhDOS of GTBT superlattice is divided in two ranges: 0–2.49 THz and 2.49–5.40 THz, which frequencies are lower than the PhDOS of GTST with two ranges 0–2.64 THz and 2.64–5.59 THz. This phenomenon leads to a little bigger heat capacity for GTBT superlattice according to the eqn (3) given below, and it have an adverse slightly impact on the decrease of the lattice thermal conductivity. The results of superlattice structures with the other three phases display the same phenomenon as the inverted Petrov phase.

In order to study how the changes of phonon group velocity and heat capacity effect on the thermal conductivity. We obtained the thermal conductivity from the calculated phonon spectrum and PhDOS. The theory we employed here is classical theories of thermal conductivity, lattice thermal conductivity is expressed as,^21^1
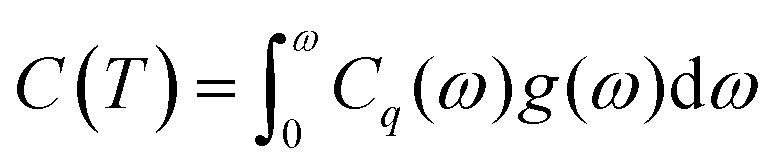
where *λ* is a set of quantum numbers specifying a phonon state, *τ*_*λ*_ is the phonon relaxation time in a given temperature, *ν* is the phonon group velocity, and *C*(*T*) is the lattice thermal capacity, who can be acquired as:2
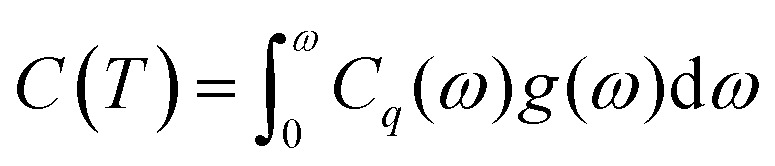
where *C*_*q*_(*ω*) is the thermal capacity for a certain wave vector *q* and frequency *ω*, and *g*(*ω*) is frequency distribution.3
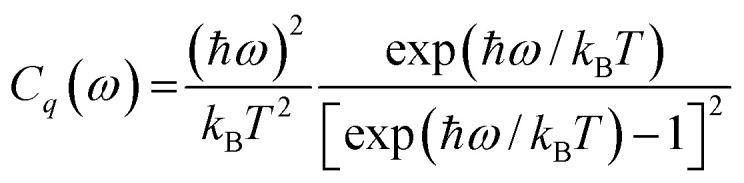


Relaxation time's inverse can be given by contributions from various scattering mechanisms:^22^4
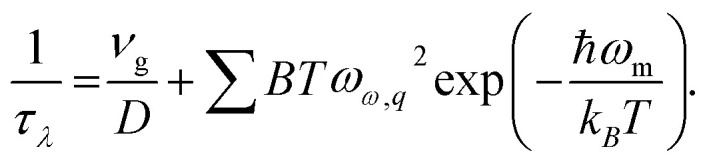


The first term stands for boundary scattering, and the second term is an empirical formula of U process. Here, *D* stands for size of ideal crystal, *ω*_m_ stands for the highest frequency in a phonon dispersion relation and *B* is a fitting parameter.

**Fig. 3:**
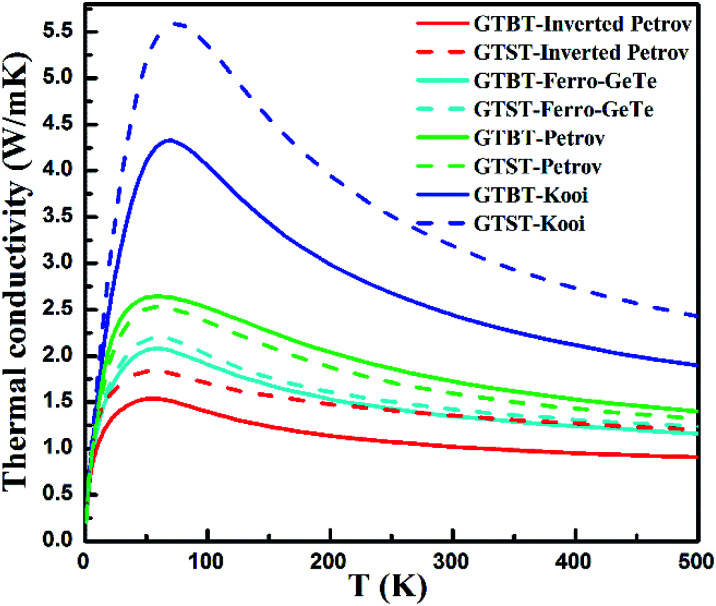
Lattice thermal conductivity of GTBT and GTST superlattice structures as a function of lattice temperature calculated.

The lattice thermal conductivities of GTBT superlattice materials are calculated based on the above calculations, as can be seen in Fig. 3. We found the lattice thermal conductivity is strongly temperature dependent, being attributed to significant contribution from U process, which is related to the phonon dispersion curves.^23^

Fig. 3 also obviously show the order of lattice thermal conductivities is Kooi > Petrov > Ferro-GeTe > inverted Petrov phase for GTBT superlattice materials, which is in line with the order of group velocity discussed in Fig. 1. This proves that while the strength of interfaces' effect becomes strong, the lattice thermal conductivities of superlattice materials will decrease. And the results of calculations demonstrate the lattice thermal conductivities of GTBT superlattice materials are smaller than GTST superlattice materials, for Kooi, Ferro-GeTe, and inverted Petrov phase, respectively. This phenomenon confirms Bi atoms in superlattices have a positive impact on reducing the lattice thermal conductivity. Meanwhile, we found the lattice thermal conductivity of GTBT superlattice with Petrov phase is a little bigger than the corresponding GTST superlattice structure. For GTBT superlattice with Petrov phase, both two Bi atoms are away from the interface, which would weaken Bi atoms' impact on the interface, and result that group velocity of GTBT with Petrov phase is only a slightly smaller than GTST with Petrov phase. Since GTBT's bigger heat capacity, Petrov phase present different change trend from other phases because of group velocity's and heat capacity's effect in combination, as shown in eqn (1). Despite this, the thermal diffusivity of GTBT superlattice with Petrov phase will still decrease, because the thermal diffusivity *α* = *k*/(*ρC*), and this is helpful to suppress the thermal crosstalk problem. This is in line with our expectation, namely proposing Bi-based phase change superlattice materials to reduce programming current and suppress the thermal crosstalk between adjacent storage units in PCM.

**Fig. 4:**
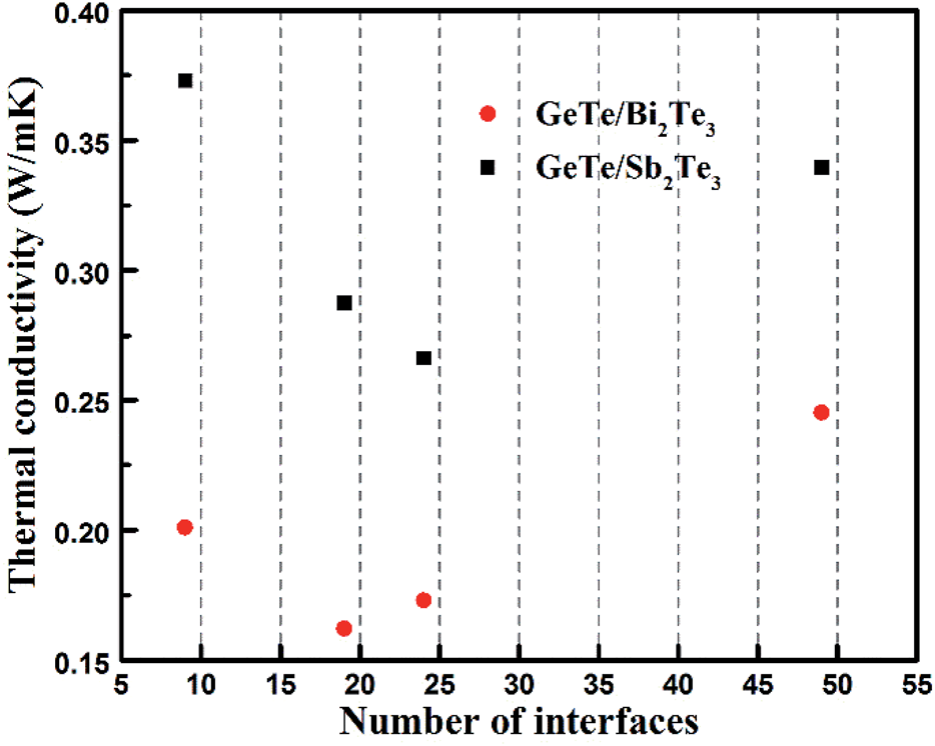
The measured thermal conductivities of superlattice-like materials with different number of interfaces (*N*) for GeTe/Bi_2_Te_3_ and GeTe/Sb_2_Te_3_. Both GTBT and GTST samples are covered with SiO_2_ layer to guarantee insulation.

To verify the above change trend of GTBT superlattice materials' lattice thermal conductivity, we measured the thermal conductivity of GeTe/Bi_2_Te_3_ superlattice-like film materials by 3ω method. The 3ω method is a well-established method for measuring the thermal conductivity of a thin film.^24^ The GeTe/Bi_2_Te_3_ superlattice-like film materials are formed by alternately depositing GeTe and Bi_2_Te_3_, and interfaces are formed between two different chalcogenide material layers. The total thicknesses of all films are kept 150 nm and the thickness ratio of GeTe to Bi_2_Te_3_ is kept at 2 : 1, the number of interfaces *N* is controlled by varying the period length of each cycle. Fig. 4 shows the measured thermal conductivities of GeTe/Bi_2_Te_3_ superlattice-like materials with total thicknesses 150 nm and different number of interfaces (*N*). It exhibits GeTe/Bi_2_Te_3_ superlattice-like materials of all numbers of interfaces show a lower thermal conductivity than the GeTe/Sb_2_Te_3_ superlattice-like materials, and reach an extremely low thermal conductivity at 0.162 W mK^−1^. This drop is in accordance with our calculated results, and proves GeTe/Bi_2_Te_3_ superlattice materials are promising candidates to reduce the thermal conductivity of phase-change materials. Additionally, we found an interesting phenomenon that a minimum thermal conductivity occurs in the process of increasing *N*, which is probably because the phonon exhibits wavelike behaviour when the period length of superlattice-like materials is shorter than the phonon mean free path, as reported by Simkin *et al.*^25^

## Conclusions

Modifying the component materials and the interfaces, we proposed Bi-based GeTe/Bi_2_Te_3_ superlattice-like materials to significantly reduce the thermal conductivity of phase-change materials. Based on DFPT method, we calculated the phonon dispersion curves and PhDOS for GTBT superlattice structures, and obtained the lattice thermal conductivity along the out-of-plane direction according to classical theories. The results revealed that the lattice thermal conductivity or thermal diffusivity of GTBT superlattices would significantly decrease due to the effects of interfaces and Bi atoms. These change trends are advantageous for PCM and in line with our expectation. Under the guidance of DFPT calculations results, we also fabricated the GeTe/Bi_2_Te_3_ superlattice-like materials and measured the thermal conductivity by 3ω method. The results on experiments show an extremely low thermal conductivity at 0.162 W mK^−1^ and in line with the theoretical calculations. Our findings prove Bi-based GeTe/Bi_2_Te_3_ superlattice-like materials being promising candidates as phase change materials with lower thermal conductivity, which is advantageous to reduce programming current and suppress the thermal crosstalk between adjacent storage units in PCM.

## Conflicts of interest

There are no conflicts to declare.

## Supplementary Material

## References

[cit1] Ovshinsky S. R. (1968). Phys. Rev. Lett..

[cit2] Matsunaga T., Yamada N., Kojima R., Shamoto S., Sato M., Tanida H., Uruga T., Kohara S., Takata M., Zalden P., Bruns G., Sergueev I., Wille H. C., Hermann R. P., Wuttig M. (2011). Adv. Funct. Mater..

[cit3] Chong T. C., Shi L. P., Zhao R., Tan P. K., Li J. M., Lee H. K., Miao X. S., Du A. Y., Tung C. H. (2006). Appl. Phys. Lett..

[cit4] Lee T.-Y., Kim K. H. P., Suh D.-S., Kim C., Kang Y.-S., Cahill D. G., Lee D., Lee M.-H., Kwon M.-H., Kim K.-B., Khang Y. (2009). Appl. Phys. Lett..

[cit5] Kim S. , Lee B., Asheghi M., Hurkx G. A. M., Reifenberg J., Goodson K. and Wong H. S. P., in Thermal disturbance and its impact on reliability of phase-change memory studied by the micro-thermal stage, 2010, p. 99

[cit6] Lee B. C., Ipek E., Mutlu O., Burger D. (2009). ACM SIGARCH Computer Architecture News.

[cit7] Burr G. W., Breitwisch M. J., Franceschini M., Garetto D., Gopalakrishnan K., Jackson B., Rajendran B. (2010). J. Vac. Sci. Technol. B Nanotechnol. Microelectron..

[cit8] Tong H., Miao X. S., Cheng X. M., Wang H., Zhang L., Sun J. J., Tong F., Wang J. H. (2011). Appl. Phys. Lett..

[cit9] Lee T.-Y., Kim K.-B., Cheong B.-k., Lee T. S., Park S. J., Lee K. S., Kim W. M., Kim S. G. (2002). Appl. Phys. Lett..

[cit10] Hafner J. (2008). J. Comput. Chem..

[cit11] Yavorsky B. Y., Hinsche N. F., Mertig I., Zahn P. (2011). Phys. Rev. B: Condens. Matter Mater. Phys..

[cit12] Goldak J., Barrett C. S., Innes D., Youdelis W. (1966). J. Chem. Phys..

[cit13] Petrov I., Imamov R., Pinsker Z. (1968). Sov. Phys. Crystallogr..

[cit14] Kooi B. J., De Hosson J. T. M. (2002). J. Appl. Phys..

[cit15] Tominaga J., Kolobov A. V., Fons P., Nakano T., Murakami S. (2014). Adv. Mater. Interfaces.

[cit16] Togo A., Oba F., Tanaka I. (2008). Phys. Rev. B: Condens. Matter Mater. Phys..

[cit17] Shaltaf R., Gonze X., Cardona M., Kremer R. K., Siegle G. (2009). Phys. Rev. B: Condens. Matter Mater. Phys..

[cit18] Hellman O., Broido D. A. (2014). Phys. Rev. B: Condens. Matter Mater. Phys..

[cit19] Chen G. (1998). Phys. Rev. B: Condens. Matter Mater. Phys..

[cit20] Chen G., Neagu M. (1997). Appl. Phys. Lett..

[cit21] Tamura S.-i., Tanaka Y., Maris H. J. (1999). Phys. Rev. B: Condens. Matter Mater. Phys..

[cit22] Balandin A., Wang K. L. (1998). Phys. Rev. B: Condens. Matter Mater. Phys..

[cit23] Klemens P. G. (1966). Phys. Rev..

[cit24] Cahill D. G., Pohl R. O. (1987). Phys. Rev. B: Condens. Matter Mater. Phys..

[cit25] Simkin M. V., Mahan G. D. (2000). Phys. Rev. Lett..

